# Serum as a Remedy for Hog-Cholera1Read before the Nebraska Veterinary Medical Association, February, 1899.

**Published:** 1899-06

**Authors:** J. D. Sprague

**Affiliations:** David City, Neb.


### SERUM AS A REMEDY FOR HOG-CHOLERA.[1]

By J. D. Sprague, V.S.,
DAVID CITY, NEB.

Since the introduction of the use of blood-serum as a therapeutic agent, and particularly since Dr. A. T. Peters applied it in the treatment of hog-cholera, stock-raisers and veterinarians have looked forward with much interest to the merits or demerits of the system.

No doubt this is of little interest to the city veterinarian, but the disease has been the source of much annoyance to those located in country districts. It is very embarrassing to be approached by a client relating his experience and describing minutely the symptoms of so common a disease, or, what is more common, to have him say "My hogs have cholera," or "My cattle are dying in the stalks,"

[1] Read before the Nebraska Veterinary Medical Association, February, 1899.

and ask you for advice in preventing further loss. I say it is very embarrassing, indeed, in such a case to try to content yourself or satisfy your client by telling him this disease is due to a living organism, a microbe, germ, etc., or that the cause is not definitely known, and the only relief to be obtained is for him to change his herd to another pasture, separate the healthy from the infected animals, disinfect the pens, etc., or, as a last resort, throw them on the market at whatever they will bring.

You are beaten out of your visit, your counsel is given without compensation, for you consider it of little value, and the farmer returns to his scene of trouble, brooding over his misfortunes and wondering what the veterinary profession is anyway if they are so ignorant as to know nothing of such diseases, or, if so, indifferent as to give it no investigation, in many cases making up his mind that where he can best use the veterinarian is where he can get along without him.

I have often heard it said that cholera only furnished the market for surplus hogs, and that a cure or prevention of the disease would be detrimental rather than beneficial to the hog raiser, on the theory that it would reduce the market price; but no doubt the price in this case would be regulated just as it is with all other products. We all know that the greatest drawback to hog raising is the uncertainty of getting the animals to market, and anything that will relieve this uncertainty would naturally be a great benefit. And as for the profession, how much better would it be if in such cases we could say, "Yes, we recognize the trouble, and by the application of such and such treatment we may be able to prevent further spread of the disease, and perhaps cure a certain percentage of the infected animals, and thereby reduce the loss to a minimum?" He not only pays your bill and looks pleasant, but it also raises you in his estimation, and likewise convinces him of the fact that the men following the profession are worthy of the name; and while there may be but little or no financial returns directly in the treatment of the hogs, yet I am quite sure that most any country practitioner can well afford to treat the hogs as well as the other animals.

It is not my intention to theorize upon the curative properties of hog-cholera antitoxin from a scientific stand-point—that is, whether or not its action is due to ptomaïns, leucomaïns, or to some peculiar action of the phagocytes, or what action it has upon the blood of the animal treated to render it immune—for bacteriologists alone are capable of dealing with such questions, and they know little enough about this particular one; but what interests us most is whether or not the use of serum in hog-cholera will be practical; and what little I say along this line will be from observations made in the use of it during the last two years. I will roughly review my use of it in the different herds:

Lot I.—100 head, free from disease at the time of inoculation, and remained so; but as we never knew of their being exposed to the infection it furnished no evidence of the efficiency of the remedy.

Lot II.—20 head, free from the disease at the time of treatment, were placed in infected pens. All remained well for two months, when one died after a few days' illness.

Lot III.—30 head, healthy hogs, treated and in ten days placed in pens where owner had lost all of his hogs three months prior. All remained healthy.

Lot IV.—Badly infected herd of 80 head; treated 40 and separated them from the balance of the herd; 10 of the treated and 25 of those not treated died.

Lot V.—45 head of pigs, generally infected, but, unknown to the owner, were placed in pens with 55 healthy hogs. In five days 2 of the 45 head died, and most of the others, with 2 of the 55 head, showed plainly symptoms of cholera. These were removed to another pen and the entire 98 head treated with serum. None died until the third day after treatment, when those of the infected lot began dying, the disease taking its regular course. This continued for several days, when I gave the infected ones another injection of serum. None died until the third day after the second injection, when they again began dying. I then repeated the inoculation, which seemed to check the disease again for three days, when they began dying, and continued until the entire 45, with the 2 of the 55, were dead. The 53 remained perfectly healthy. About ten days after the first treatment one of the healthy animals, which had become slightly crippled in some way, was placed with the sick ones. This remained well, and after the sick ones had all died it was replaced with the original bunch.

Lot VI.—Treated 60 head in feed-lot containing about 150 head. A few were dying with symptoms of cholera. Very few of the treated animals died, but owing to many of the original hogs being taken out and replaced by others the percentage could not be obtained.

Lot VII.—18 head that were left of a bunch of about 50, the others having died with cholera. These were each given one dose of serum. The second day about one-half of them were given a second dose; the third day those presenting the worst symptoms were again treated, and on the fourth day the same. None died until about one week after the treatment. At this time some of them showed symptoms of being worse, and 4 died, 14 making a nice recovery.

You will notice that in some cases I have been repeating the doses. In the next experiment, Lot VIII., I have taken extra care to ascertain if there be any benefit in so doing, selecting subjects from an infected herd in which the symptoms were well marked. These were separated from the balance of the herd and treated once daily until showing improvement or dying. Treated 17 head, 4 of them dying. Four of the herd not treated also died. Some required only two doses, while one took ten. Two of the 4 which died, died with symptoms of cholera, one of pneumonia, and one apparently from septicæmia. This last one was the subject given ten doses of serum.

The tenth day after beginning treatment the symptoms of cholera had disappeared, but the subject did not improve as it should, but instead continued to grow thinner and weaker until death. It is my opinion that death in this case was due to too much serum, but had it not been used death would probably have been produced by cholera. As I have said, one of this lot died of pneumonia, and it is in such cases as this, where there is a development of some local affection, that the use of serum is most often condemned, for hog-cholera antitoxin will not cure these complications.

It is a well-known fact that one of the characteristics of this disease is its liability to affect any organ of the entire body. This peculiarity is perhaps due to the greatly contaminated condition of the blood, together with the functional sympathy of the internal organs; and I have often thought, too, there is a tendency for the bacilli to accumulate at the seat of any local hyperæmia.

Through this condition of the blood the function of some organ becomes perverted, which may, by functional sympathy, derange the function of some other organ. This condition may then be followed by congestion and even inflammation of the parts, which, no doubt, furnishing a more suitable soil for their propagation, causes the bacilli to accumulate and multiply very rapidly, creating or increasing the already existing local inflammation, thereby producing pneumonia, enteritis, nephritis, or whatever it may be, depending upon the location. After almost any of these local affections have become well established the germ of hog-cholera may be destroyed and the animal succumb from the local trouble. For this reason serum in many cases fails to have the desired effect. I mention this to show the necessity of sanitation and early treatment in order to be successful, for any unsanitary surroundings, with the neglect of treatment for a few days, may be all that is necessary for the development of such complications, which might have been avoided.

In treating an outbreak of cholera I think it is best to begin as soon as you discover the disease. Give each animal of the herd one inoculation. Separate the sick from the balance of the herd, and repeat the treatment upon these and any others that may show acute symptoms, once a day for two to four days, depending upon the severity of the symptoms. If the herd is very badly infected I think it is best to change the entire herd to another yard. The dose I have been giving is about 10 c.c. to one-hundred-pound hogs in large hogs, and never giving less than that in smaller ones. I think it best to insert the needle deeply into the inguinal region, for when injected just under the skin one-third to one-half of the dose will return through the opening made by the needle. I have never seen an abscess form in this region. While the results of some experiments are not as satisfactory as we desire, yet I believe if the serum be properly administered, and we exercise as much judgment and use the the same precaution in the treatment of this disease as we do of others, we will have very favorable returns and save perhaps 80 per cent. of the infected herds.

Some of my statements may not concur fully with the ideas of Dr. Peters and some other authorities, but I wish it understood that they are only my opinions, and I do not pose as an authority. I have made some other experiments in the last two weeks, among them repeating inoculation in healthy animals as a preventive measure, but am sorry sufficient time has not elapsed to observe their effects.

